# Treatment of the Deciduous Dentition

**Published:** 1912-03-15

**Authors:** N. S. Hoff


### TREATMENT OF THE DECIDUOUS DENTITION.

BY N. S. HOFF, D.D.S.

Because of the fact that the first or deciduous denture has so short an existence, and it usually serves the purpose of mastication even though more or less impaired, most parents give little or no attention to their childrens teeth until forced to do so when they become so diseased as to be painful, and even then they are satisfied with such treatment as shall relieve the discomfort and avoid the complaining of the child. It is only in recent times that we have begun to realize the importance of maintaining these teeth intact throughout the full period of their normal existence. As with the permanent teeth, the great foe to their natural integrity and function begins with caries. In some cases these teeth become extensively carious within a few months after their emergence from the gum tissue, and at the age of from one to three years, when the child is so tender and easily hurt by the usual dental ministrations, it is an exceedingly difficult matter to provide suitable prophylactic remedies, and even more so to make adequate repair treatment.

Almost innumerable suggestions have been made to prevent and repair carious destruction of the deciduous teeth. Where the discoloration of the teeth produced by nitrate of silver is not objectionable, this makes a very satisfactory prohibitive medicinal remedy and when it is properly applied it is usually effective. For its application the cavity should be excavated so as to free it from all the carious debris possible and the silver nitrate should be applied to a dry cavity in a completely isolated tooth. The silver should be in the form of a saturated aqueous solution. It should be applied so as to be retained in the tooth cavity for at least ten minutes, while the direct sun rays are directed into the cavity, so as to decompose the silver into a black deposit penetrating the decomposed dentine. This silver salt will permeate the dead and disorganized tooth substance converting it into an immune and insoluble material which will arrest all fermentable processes for months and years in some cases. Care must be exercised not to place it directly on the pulp tissue or even on near exposures or the vitality of the pulp will suffer.

Another method of treating especially incipient caries, is that suggested by Dr. G. V. Black in his work on operative dentistry, vol. I, page 248, where he suggests separating the incisal teeth that become carious in the first degree by filing away the infected area and polishing the abraded dentine and then saturating it with silver nitrate and repeating the treatment on successive periods until a hard and shining surface is obtained. His claim is that the food incursions in mastication will keep these surfaces free from bacterial deposits and caries will not recur. He applies the filing and separating to the molar teeth on the same principle, except that the proximal contacts are preserved, for a limited area only to prevent the teeth from coming into closer contact than normal which would tend to stop the interstitial bone growth of the jaw and alveolus. The disadvantages of this treatment is that it mutilates the teeth, is somewhat painful and the teeth are badly discolored.

*Prophylaxis polishing*, where practicable, is the surest and most agreeable treatment, as it is not only successful, but it inculcates most valuable lessons as to the value of the teeth and the necessity for constant application of an effective mouth toilet. Its only disadvantage is that it must be repeated at frequent intervals for indefinite time, and it can not be applied where any considerable degree of caries is present in active form.

The restoration of lost tooth stucture by various filling materials is effective but in most cases painful and difficult of application to young and sensitive children; but for second and third degree caries there seems to be no other adequate remedy, as in most of these cases the tooth is rendered inefficient in function and must be restored to normal form by a suitable and insoluble material. While there are difficulties to be overcome in the treatment of limited carious affections, there are almost insurmountable obstacles to overcome when the caries advance sufficiently to expose and infect the pulp of the tooth. A long line of disturbances extending from mere odontalgia to alveolar necrosis which demand a variety of therapeutic skill that tests our ingenuity to overcome.

The temptation is very great when the pulp becomes involved, even to the extent of accidental exposure, to extract the tooth; especially when only a few months are likely to intervene before the permanent tooth should take its place, and most dentists extract deciduous teeth having alveolar abscesses without raising the question of the advisability of such a procedure. There is much to be said in favor of extraction in such cases, especially when a prolonged or painful treatment is necessarily involved and there is a possibility of injury to the calcification organ of the permanent tooth. But the loss of the function of even a single deciduous tooth has so great an influence on alveolar development that there can be no question as to the right practice in such cases. A good general rule to follow is not to extract any deciduous tooth until its successor is ready to replace it. This rule will necessarily imply more or less treatment of childrens teeth, for the reason that we do not have all children sufficiently under surveillance to prevent the progress of caries to the destructive extent.

The treatment of deep-seated caries which has not yet involved the pulp is comparatively simple and involves no unusual treatment and needs no further comment here. When there is a near approach to exposure because of the extraction of the calcified tissue immediately covering the pulp, there is necessarily infection in contact with the pulp organ if it has not already produced a lesion. All such conditions necessarily involve disinfective treatment, which must be of such character as not to produce excessive pain. Operative disinfection can only be extended to the removal of the insensible and disorganized structure, and to the shaping of the cavity margins to enable the hermetical sealing of a non-irritant disinfectant in the cavity for sufficient time to accomplish this object. For this purpose a mixture of zinc oxide and thymol made into a paste with a drop of oil of cloves may be gently applied and covered with a thin piece of asbestos paper, or a metal cap, and the cavity then filled with Calxine Cement, which may be left for two or three weeks, when a portion of the cement can be removed and a more durable filling applied.

*Infected and Suppurating Pulps.* The most troublesome cases for treatment are abscessed deciduous teeth and care should be taken that alveolar abscesses never develop from infected pulps in deciduous teeth. The problem of devitalizing or surgically removing an infected pulp of a deciduous tooth is many times a difficult one to handle. The possibility of serious injury to the apical tissues from the use of arsenical pastes almost excludes the application of this kind of treatment; although it may be safely used in those teeth which have not a long time to remain in place until replaced by the permanent teeth. If reasonable care is used in applying the arsenical treatment and it be not left too long in the tooth it should not produce the excessive action feared on the enamel organ of the permanent tooth or the contiguous tissues. It certainly is much to be preferred that the deciduous pulp be entirely removed and the canal aseptically treated and filled, rather than to develop an alveolar abscess, with its possible sequellae of necrosis.

If the pulp be not seriously infected anesthetization either by the pressure or injection method should be practicable if the patient can be controlled; and the subsequent treatment and filling be accomplished with little or no danger of septic results. It is not necessary and generally not desirable that the durable root filling required in the permanent teeth be used. Root fillings that can be inserted in the paste form to subsequently harden, or those in the form of readily fused waxes will generally be all that are required to tide over the brief period of their probable existence. Our preference is for a soft wax in which is incorporated non-irritating and enduring antiseptics to be inclosed with a good cement. In some cases it becomes necessary to make use of the deciduous teeth in spreading the arches in preparation for the incoming permanent teeth. In such event no amalgam or oxyphosphate of copper filling should be placed in the teeth, as a disagreeable electrolysis is liable to occur because of the metallic bands, arches and ligatures used in the orthodontic procedures.

If the teeth are already abscessed and any considerable period must elapse before the permanent tooth can be expected, the abscess should be carefully treated and the root filled as indicated above, if this can be done so as to have a healthy condition at the apical region. Otherwise it would be much better to extract the offending tooth and place some form of a splint on the adjoining teeth that the space may be preserved for the permanent tooth. This may not always be practicable as several deciduous teeth may be lost at the same time and no sufficient abutting teeth be available for the attachment of the bridge splint. These unfavorable abscess conditions are so frequent that we are many times tempted to extract the diseased teeth that seem almost hopeless so far as successful treatment is concerned. There can be no doubt that a healthy environment for the developing permanent tooth is important and that we are justified in extracting incurable teeth to secure such a condition; but we should always understand that this necessarily interferes with the prompt and natural eruption of the permanent tooth, and may produce conditions requiring orthodontic interference later on. For prophylactic reasons therefore we should by all means prevent all diseases of the deciduous denture that will necessitate the premature loss of a single tooth. The future preventive dentistry will concern itself more with the care of the deciduous teeth, and the eruption of the permanent teeth into normal or typical relations than has heretofore been thought necessary.

